# Sensitivity of *Salmonella* serovars and natural microflora to high-pressure pasteurization: Open access data for risk assessment and practitioners

**DOI:** 10.1016/j.dib.2018.09.071

**Published:** 2018-10-04

**Authors:** Abimbola Allison, Aliyar Fouladkhah

**Affiliations:** Public Health Microbiology Laboratory, Tennessee State University, Nashville, TN, United States

**Keywords:** High-pressure pasteurization, *Salmonella* serovars, Natural microflora, Elevated hydrostatic pressure

## Abstract

Industrial adoption of high-pressure processing is gaining importance and momentum as an alternative method to traditional utilization of antimicrobials and heat-based pasteurization. This indicates the need for extensive validation studies and available data for feasible and efficacious adoption of the technology by practitioners and the private industry. Current dataset is obtained utilizing elevated hydrostatic pressure of 35 to 380 MPa for time intervals of 0 (untreated controls) to 10 min, for decontamination of mesophilic background microflora and inoculated *Salmonella* in orange juice [1]. This open accessed data could be incorporated as part of risk assessment analyses for mitigating the risk of non-typhoidal foodborne salmonellosis by public health practitioners. It could also be utilized to validate the efficacy of elevated hydrostatic pressure against *Salmonella* serovars and background microflora in food manufacturing.

**Specifications table**

**Table:** 

Subject area	Public Health Microbiology
More specific subject area	Microbial Food Safety
Type of data	Microsoft Excel Workbook (.xlsx extension)
How data was acquired	Inactivation of five-strain habituated *Salmonella* serovars and mesophilic natural microflora was achieved using elevated hydrostatic pressure of 35 to 380 MPa (5 K to 55 K PSI) at a controlled temperature of 25 °C. The pressure treatments were conducted in PULSE reaction tubes by Barocycler Hub440 unit (Pressure BioScience Inc., South Easton, MA, USA). The temperature was controlled using a stainless steel jacket surrounding the pressure chamber, connected to a circulating water bath (VWR International, Radnor, PA, USA). Temperature and hydrostatic pressure were monitored and recorded using HUB PBI 2.3.11 software.
Data format	Raw data
Experimental factors	The data is consists of two biologically independent repetitions, each considered as a blocking factor in a complete randomized block design. Each block further contains three replications (total of six replications) and each replication is a mean of two microbiological repetitions. The data contains inactivation of 5-strain habituated *Salmonella* serovars and natural microflora of orange juice. The detection limit of this study was 0.30 log Colony Forming Units (CFU) per mL.
Experimental features	This dataset is derived by exposing habituated inoculated *Salmonella* serovars and natural microflora of orange juice to elevated hydrostatic pressure. Microbial counts were obtained after neutralization of samples on a media supplemented with yeast extract to enhance the recovery of injured cells.
Data source location	Data was collected in Public Health Microbiology Laboratory of Tennessee State University in Nashville, Tennessee.
Data accessibility	The dataset could be accessed at Harvard Dataverse public repository available at: https://doi.org/10.7910/DVN/TSBJ0V [Accessed September 11, 2018]. Publication associated with this data is also available and open access [1].

**Value of the data**

• The data show decontamination efficacy of elevated hydrostatic pressure against a pathogen and natural microflora that could be of importance for public health practitioners and private industry.
• The data demonstrate the sensitivity of *Salmonella* serovars to high-pressure pasteurization that could be utilized in risk assessment analyses for mitigating the burden of salmonellosis associated with consumption of juices.
• The data demonstrate that use of elevated hydrostatic pressure at 380 MPa could lead to reductions of the pathogen and background flora in levels comparable to the traditional thermal processing of the products and thus could be incorporated as a control measure in a food safety management-based system for assuring microbiological safety of the product.
• The current data could be utilized for further calculations of linear and non-linear inactivation indices such as D- and k_max_ values for comparing decontamination efficacy of elevated hydrostatic pressure to other thermal and non-thermal practices.

## 1 Data

To obtain the current data, the hydrostatic pressure of 35 to 380 MPa (5 K to 55 K pounds per square inch [PSI]) had been applied at 0 (*i.e.* control) to 10-time intervals for inactivation of the inoculated pathogen as well as natural background microflora. Current dataset exhibits utilization of moderate levels of elevated hydrostatic pressure since higher levels of pressure-based pasteurization could lead to higher cost associated with increased maintenance and decreased life of pressure vessels used during manufacturing [2]. The high-pressure processing unit utilized for obtaining the current data has a chamber size of 16 mL with the chamber surrounded by a stainless steel water jacket mechanically linked to a refrigerated circulating water bath (VWR International, Radnor, PA, USA) for precise maintenance of the temperature during processing. For monitoring temperature, two k-type thermocouples (Omega Engineering Inc., Norwalk, CT, USA), were inserted inside the wall of the chamber, secured with thermal paste (Model 5 AS5-3.5G, Arctic Silver, Visalia, CA, USA) and connected to pressure processing software (HUB PBI 2.3.11 Software, Pressure BioScience Inc., South Easton, MA, USA). Pressure transmission medium used in this study was distilled water (total dissolved solids < 30 ppm). The processing chamber was purged before each treatment for residual air removal from the chamber׳s headspace. The treatments were conducted in no-disk PULSE tubes (Pressure BioScience Inc., South Easton, MA, USA) containing 1.5 mL of habituated inoculum and the food vehicle (orange juice, pH range of 3.79 ± 0.2 to 4.14 ± 0.3). The PULSE tubes were then subjected to hydrostatic pressure treatments of 0, 35 MPa (5K PSI), 103 MPa (15K PSI), 241 MPa (35K PSI) and 380 MPa (55K PSI), with a hold time of 1, 2, 4, 8, and 10 minutes in addition to untreated controls (Time-0). The dataset is uploaded onto Harvard Dataverse public repository, as Microsoft Excel Workbook (.xlsx extension), and is available at: 10.7910/DVN/TSBJ0V [Accessed October 12, 2018].

## 2 Experimental design, materials, and methods

### 2.1 Experimental design and statistical analyses

**Fig. 1:**
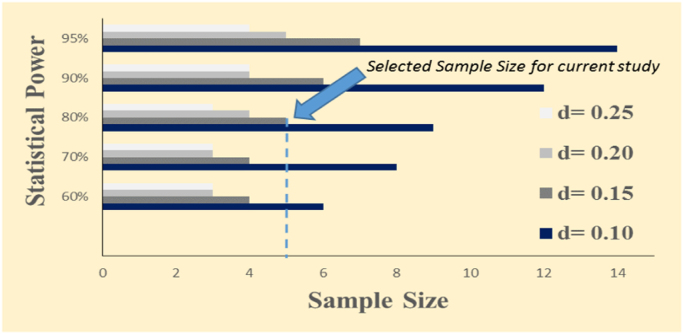
Statistical power and sample size calculation at type I error level of 5% (α= 0.05) for various detectable differences (d, log CFU/mL). Calculations derived from *Proc Power* command of SAS_9.2_ (SAS Institute, Cary, NC), based on the mean (4.52 log CFU/mL) and standard deviation (0.07) of preliminary trials (n = 5) using 5-strain mixture of *Salmonella* serovars at Public Health Microbiology Laboratory of Tennessee State University.

An *a priori* power analysis was conducted using *Proc Power* of SAS_9.2_ (SAS Institute, Cary, NC, USA.), based on existing preliminary data (data not shown) at statistical power of 80%, type one error level of 5%, and for detecting mean difference of 0.15 log CFU/mL as statistically significant. At the above-mentioned levels of confidence and power, a sample size of at least 5 samples per time/pressure was selected Fig. 1. To meet and exceed this calculated sample size, each experiment was performed in two biologically independent repetitions, each considered as a blocking factor in a complete randomized block design. Each block then consisted of three replications per time/pressure treatment, and each analysis was further conducted in two microbiological repetitions. Two separate studies were conducted for generating data associated with inactivation of 5-strain *Salmonella* serovars and decontamination of mesophilic background microflora.

### 2.2 High-pressure pasteurization

Hydrostatic pressure (Barocycler Hub440, Pressure Bioscience Inc., South Easton, MA, USA) of 35 to 380 MPa (5 to 55 K PSI) was investigated at 0 (untreated control) to 10 min time intervals for inactivation of the inoculated habituated pathogen and mesophilic background microflora. Current data exhibits a moderate intensity of elevated hydrostatic pressure that is typically associated with lower processing cost, equipment maintenance, and increased pressure vessels life in food manufacturing [2]. As previously mentioned, the high-pressure pasteurization unit used for generating the current data has a chamber size of 16 mL with the chamber surrounded by a stainless steel water jacket connected to a circulating refrigerated water bath (VWR International, Radnor, PA, USA) for precise control of the temperature. For monitoring the temperature, two k-type thermocouples (Omega Engineering Inc., Norwalk, CT, USA), were inserted inside the chamber wall, and then secured with thermal paste (Model 5 AS5-3.5G, Arctic Silver, Visalia, CA, USA). The thermocouples were connected to the unit׳s software (HUB PBI 2.3.11 Software, Pressure BioScience Inc., South Easton, MA, USA). The pressure transmission fluid used for generating the current data was distilled water (total dissolved solids < 30 ppm), prior to each analysis the chamber was purged for removal of residual air in chamber׳s headspace. The reported processing time values exclude the time for pressure increase (3 s) and exclude the release time (1 s), and were monitored using the Barocycler mode of HUB PBI 2.3.11 software (Pressure BioScience Inc., South Easton, MA, USA). As previously explained, the treatments conducted inside no-disk PULSE tubes (Pressure BioScience Inc., South Easton, MA, USA) containing 1.5 mL of orange juice inoculated with habituated *Salmonella* serovars. The PULSE tubes were then subjected to hydrostatic pressure treatments of 0, 35 MPa (5K PSI), 103 MPa (15K PSI), 241 MPa (35K PSI) and 380 MPa (55K PSI), with a holding times of 1, 2, 4, 8, and 10 min in addition to untreated controls (Time-0).

### 2.3 Bacterial inoculum preparation

For the inoculation study, a five-strain mixture of habituated *Salmonella* serovars (ATCC^®^ numbers 13076, 8387, 6962, 9270, 14028) was used. Strains were existing in the Public Health Microbiology Laboratory and were originally acquired from American Type Culture Collection (Manassas, VA, USA). These selected strains all belong to *enterica* species of Salmonellae and represent sub-species of Enteritidis, Montevideo, Newport, Anatum, and Typhimurium, respectively. These sub-species were selected based on previous screening studies in acidified foods [3] and public health significance of the serovars. The five selected sub-species belong to top 10 dominant *Salmonella* serovars of public health importance based on a 1998–2014 epidemiological study of United States Department of Agriculture [4]. A validated decontamination plan using these pathogens could assist the stakeholders to meet the regulatory requirement of Hazard Analysis and Risk-Based Preventive Controls of the Food Safety Modernization Act in the United States or comparable global regulatory requirements [5]. The pathogen mixture was prepared based on the method delineated in our recent open access publication and represent serovars of public health significance to plant-based commodities and those zoonosis strains that could lead to contamination of plant-based foods [1]. In short, each individual strain was grown planktonically in Tryptic Soy Broth supplemented with yeast extract (TSB+YE) to minimize the acid stress of the pathogen during multiplication. After incubation for 20–24 h at 37 °C, a 100 μL of the above-mentioned overnight suspension were then individually sub-cultured into TSB+YE and re-incubated for 20–24 h at 37 °C. For each individual strain, cells from the sub-cultured overnight suspension (2000 μL aliquot) were harvested using centrifugal force at 6,000 RPM (3,548 g, for 88 mm rotor) for 15 min (Model 5424, Eppendorf North America, Hauppauge, NY, USA; Rotor FA-45-24-11). For further removal of sloughed cell components, excreted secondary metabolites, and growth media, the cells were re-suspended in 2 mL Phosphate-Buffered Saline (VWR International, Radnor, PA, USA) and re-centrifuged individually using the above-mentioned time and intensity. To further improve the external validity of this inoculation study, each strain was then individually habituated in sterile orange juice for 72 h at 4 °C to allow acclimatization of the pathogen to low temperature and intrinsic factors of the product prior to the experiment. Habituated cells were then vortexed (Model Vortex-2 Genie, Scientific Industries, Bohemia, NY, USA) for 60 s, then composited into a five-strain habituated mixture of the pathogen. The cell preparation was conducted to achieve pathogen cell density of 8.5 log CFU/mL prior to habituation, and for yielding a target microbial density of 7.5 log CFU/mL after the 72-h habituation.

### 2.4 Microbiological and pH analyses

To assure neutralization of the product acidic intrinsic condition, prior to microbiological analyses and after each treatment, samples were neutralized using D/E neutralizing broth (Difco, Becton Dickinson, Franklin Lakes, NJ, USA). In order to assure maximum recovery of the injured cells, Tryptic Soy Agar supplemented with 0.6% yeast extract was selected based on conducted preliminary trials (data not shown). The neutralized microbial suspension after treatment or for untreated controls were then 10-fold serially diluted in Maximum Recovery Diluent (Difco, Becton Dickinson, Franklin Lakes, NJ, USA). For enumeration of the pathogen and mesophilic natural microflora counts, plates were incubated at 37 °C for 48 h, were then manually counted and converted to log values. The pH of all enumerated samples was measured after treatments using a digital pH meter calibrated at pH levels of 4, 7 and 10 prior to analyses (Mettler Toledo AG, Grelfensee, Switzerland).
