# Supplementary material for: Sensitivity of Salmonella serovars and natural microflora to high-pressure pasteurization: Open access data for risk assessment and practitioners
Source: Data Brief. 2018 Oct 4;21:480–4. doi: 10.1016/j.dib.2018.09.071 (PMC6198022; doi:10.1016/j.dib.2018.09.071)
Supplement: Supplementary file 1 — Supplementary material [file mmc1.pdf]

Conflicts of Interest Statement

Manuscript title: Sensitivity of Salmonella serovars and Natural Microflora to High-pressure pasteurization: Open Access Data for Risk Assessment & Practitioners

The authors whose names are listed immediately below certify that they have NO affiliations with or involvement in any organization or entity with any financial interest (such as honoraria; educational grants; participation in speakers' bureaus; membership, employment, consultancies, stock ownership, or other equity interest; and expert testimony or patent-licensing arrangements), or non-financial interest (such as personal or professional relationships, affiliations, knowledge or beliefs) in the subject matter or materials discussed in this manuscript.

Author names:

Aliyar Fouladkhah, PhD, MPH

Abimbola Allison

The authors whose names are listed immediately below report the following details of affiliation or involvement in an organization or entity with a financial or non-financial interest in the subject matter or materials discussed in this manuscript. Please specify the nature of the conflict on a separate sheet of paper if the space below is inadequate.

Author names:

This statement is signed by all the authors to indicate agreement that the above information is true and correct (*a photocopy of this form may be used if there are more than 10 authors*):

Author's name (typed)	Author's signature	Date
Aliyar Fouladkhah, PhD, MPH	[signature]	9/11/2018
Abimbola Allison	[signature]	9/11/2018
